# Olfactory Responses of *Asproparthenis punctiventris* Germar to Leaf Odours of Amaranthaceae Plants

**DOI:** 10.3390/insects15040297

**Published:** 2024-04-22

**Authors:** Elisabeth H. Koschier, Lena Dittmann, Bernhard Spangl

**Affiliations:** 1 Department of Crop Sciences, Institute of Plant Protection, University of Natural Resources and Life Sciences, Gregor-Mendel-Strasse 33, 1180 Vienna, Austria; 2 Department of Landscape, Spatial and Infrastructure Sciences, Institute of Statistics, University of Natural Resources and Life Sciences, Peter-Jordan-Strasse 82/I, 1190 Vienna, Austria; bernhard.spangl@boku.ac.at

**Keywords:** *Bothynoderes punctiventris*, food plants, four-arm olfactometer, *Beta vulgaris*

## Abstract

**Simple Summary:**

Insect pests can be controlled by manipulating their behaviour, which can be achieved by understanding the cues they use to find their food plants. In this study, we explored how the sugar beet weevil, *Asproparthenis punctiventris*, responds to the odours of various food plants. Using an olfactometer, we tested the reactions of adult weevils to the volatile leaf odours of different food plants, including sugar beet and chard. The results showed that both male and female weevils were attracted to the leaf odours of young sugar beet and chard plants. However, only males responded positively to the odour of the garden orache, while there was no response to the odours of fat hen or the common amaranth. This suggests that the weevil uses leaf odours to locate sugar beet and other food plants. Knowledge of the olfactory responses of this pest can provide a basis for improved monitoring or mass trapping strategies.

**Abstract:**

Understanding the stimuli used by insect pests to find their food plants can be a first step towards manipulating their behaviour and, thus, controlling them. We investigated the responses of the sugar beet weevil *Asproparthenis punctiventris* (Coleoptera: Curculionidae) to the volatile leaf odours of its food plants, including *Beta vulgaris* subsp. *vulgaris* (Altissima and Cicla groups), *Atriplex hortensis*, *Chenopodium album*, and *Amaranthus retroflexus*, in a four-arm olfactometer. A bioassay procedure was developed, and the frequency of visits and time spent by adult weevils in the quadrant of the olfactometer with leaf volatiles was recorded, as was their first choice of quadrant. Females and males were equally attracted to the leaf odours of young *B. vulgaris* subsp. *vulgaris* plants, i.e., sugar beet and chard, as indicated by the overall picture of the behavioural parameters analysed. Males, but not females, responded positively to the leaf odour of the garden orache (*A. hortensis*), and no response was observed when the weevils were tested with the leaf odours of fat hen (*C. album*) or common amaranth (*A. retroflexus*). These results suggest that *A. punctiventris* uses leaf odours to locate sugar beet and other food plants. Knowledge of the olfactory responses of this pest can provide a basis for improved monitoring or mass trapping strategies.

## 1. Introduction

The sugar beet weevil *Asproparthenis* (*Bothynoderes*) *punctiventris* (Coleoptera: Curculionidae) is an important pest of sugar beet (*Beta vulgaris* subsp. *vulgaris* Altissima group) crops and is widespread in Southeastern Europe, Turkey, and Eurasian countries [1,2,3].

The main damage to sugar beet is caused by the activity of the adult weevils, which occurs shortly after they emerge from their overwintering sites in the soil. During the subsequent maturation feeding phase, females and males consume sugar beet seedlings or young plants, resulting in their destruction [4,5,6]. The feeding activity can damage entire fields, often requiring reseeding [7,8].

Recent reports from Poland, Eastern Croatia, and Hungary indicate that the pest has spread further towards Central Europe and become increasingly destructive [2,7,9]. In Eastern Austria, a mass outbreak led to the loss of almost a quarter of the total sugar beet area in 2018, and the sugar beet weevil continued to cause economic damage in subsequent years [8,10]. One of the reasons for the increase in damage is that high temperatures and an increasing number of summer days, i.e., days with daily maximum temperatures of 25 °C or higher, during the activity period of the parent generation and the egg and larval phase are favourable to the population development of *A. punctiventris* [11], conditions that have become more frequent in recent years due to climate change. Another is the lack of effective insecticides; The European Union recently banned the use of neonicotinoids for seed treatment because of the risk they pose to bees. The systemic mode of action of these insecticides has protected seedlings and young plants from the sugar beet weevil, at least in years with moderate infestations [10,12].

Entomopathogenic nematodes [7,13] or entomopathogenic fungi, such as *Metarhizium brunneum* [14], could be used for the biological control of *A. punctiventris*. Although these initial studies have confirmed the efficacy of the natural antagonists, more research is needed before they can be used in practice. Another approach is the mass trapping of adult sugar beet weevils in sugar beet fields from the previous year, i.e., their overwintering sites, in early spring. In extensive field trials, Tomasev et al. [15] and Drmic et al. [16] tested this method with the aggregation attractant Grandlure III-IV in traps and were able to successfully reduce pest populations, albeit only in years of moderate infestation. Toth et al. [17,18] previously found that these compounds attracted both sexes of *A. punctiventris* in field trapping experiments, but this has never been verified in the laboratory, e.g., in olfactometer experiments.

To date, there is limited information on the chemical ecology or host plant location of *A. punctiventris*, with most relying on field observations. In 1936, Eckstein [1] described that “sensory input on the antennae” of adult weevils is responsible for locating sugar beet plants after overwintering. Similar observations were reported by Auersch [19] on weevils wandering on the ground during this phase. However, neither author discriminated between males and females. The two sexes may respond differently to plant odours, as the males leave their overwintering sites in the soil earlier than the females [19,20] and therefore search for food plants before the females emerge. Phytophagous insects usually use plant volatiles to locate suitable hosts for feeding or oviposition [21], which is particularly true for species with an oligophagous feeding pattern [22], such as *A. punctiventris*. Dittmann et al. [23] showed that the adults feed mainly on *B. vulgaris* subsp. *vulgaris* and few other Amaranthaceae species, such as the garden orache *Atriplex hortensis*, the fat hen *Chenopodium album*, and the common amaranth *Amaranthus retroflexus*. Plant species of the Polygonaceae family were hardly or not at all fed on. In this comparison of food consumption, adult weevils were tested individually in petri dishes with leaves of different food plants. However, this experimental design did not allow a distinction to be made between whether the leaves were recognised by their odour and/or whether they were accepted as food on direct contact.

Whether *A. punctiventris* uses volatile leaf odours to locate sugar beet and other suitable food plants after emergence from the overwintering site has not been investigated so far. This knowledge can be a first step towards manipulating the behaviour of this pest and, thus, controlling it. Therefore, the aim of this study was to develop an olfactometer bioassay procedure suitable for quantifying the responses of *A. punctiventris* to olfactory stimuli to compare the responses of female and male sugar beet weevils to the leaf volatiles of different Amaranthaceae food plants.

## 2. Materials and Methods

### 2.1. Insects

In April and May of 2021 and 2022, *A. punctiventris* adults were collected shortly after emerging from their overwintering sites in the soil of winter wheat fields, which were previously used for sugar beet cultivation and had been infested with sugar beet weevils. The collection was carried out at different locations in the Tullnerfeld region in Lower Austria. The insects were captured using pitfall traps (Csalomon^®^, Plant Protection Institute, Centre for Agricultural Research, HAS, Budapest, Hungary) that were baited with the aggregation attractant Grandlure III-IV (Bedoukian Research Inc., Danbury, CT, USA). In the laboratory, the weevils were subjected to artificial overwintering conditions maintained at 5 ± 1 °C and 80 ± 5% relative humidity in darkness. A total of 48 to 72 h before the experiments, the individuals were separated by sex. Unmated females and males were kept in separate plastic boxes with a bottom layer of coarse quartz sand without food in a climate chamber at 20 ± 1 °C, 78 ± 5% relative humidity, with a 14:10 h (L:D) photoperiod.

### 2.2. Plant Material

The food plant species tested were sugar beet (*Beta vulgaris* L. subsp. *vulgaris* Altissima group, cv. Blandina, KWS Austria Saat GmbH, Vienna, Austria), chard (*Beta vulgaris* L. subsp. *vulgaris* Cicla group, cv. Lucullus, Floraself, Hornbach Baumarkt AG, Germany), fat hen (*Chenopodium album* L., Staphyt Austria GmbH, Rohrau, Austria), garden orache (*Atriplex hortensis* L., Magic Garden Seeds GmbH, Regensburg, Germany), and red-root pigweed (*Amaranthus retroflexus* L., Staphyt Austria GmbH, Rohrau Austria). All plants were raised from seeds and grown in pots measuring 6.5 × 6.5 × 9 cm. The substrate mixture used was peat: quartz sand: expanded clay in a proportion of 2:1:1. Depending on their specific temperature requirements, the plants were grown either in a walk-in climate chamber or in the greenhouse. They were regularly watered with tap water.

Approximately one hour before the start of each experiment, leaves of the respective test plant were harvested and placed in a 50 mL beaker filled with water. The quantity of leaves was determined based on the leaf area equivalent to 20 sugar beet leaves ranging in size between BBCH stages 14 to 19 (4 to 9 or more leaves unfolded). This leaf quantity filled the airspace of the glass jar (0.75 litres) into which the beaker with the leaves was placed (see below).

### 2.3. Olfactometer Bioassay

A four-arm olfactometer of the basic design described by Vet et al. [24] was used to investigate the olfactory responses of *A. punctiventris* adults toward the leaf odours of food plants. The olfactometer, measuring 30 × 30 × 3.2 cm, consisted of a base plate, a central structure made of white polyethylene, and a transparent cover plate made of acrylic glass. White filter paper was attached to the floor of the star-shaped arena, which was situated in a dark room at 23 ± 1 °C and illuminated by a halogen lamp placed over its centre. A hole (1.8 cm in diameter) cut in the centre of the cover plate allowed the weevils to be introduced using a pair of tweezers, as well as to be closed using a silicone stopper with a silicone tube attached. Air was drawn through this silicone tube from the centre of the cover plate using a laboratory pump and regulated using a flowmeter (Q-Flow, Vögtlin Instruments, Muttenz, Switzerland), ensuring an airflow of 250 mL/min at each of the 4 corners, which served as inlets into the arms of the arena. Before entering the arena, the airflow passed through gas washing bottles filled with activated charcoal for purification. The purified air was then led into glass jars (0.75 litre) covered with fitted acetate sheets as lids and sealed with tape. One jar contained leaves from the plant to be tested in a 50 mL beaker filled with tap water, serving as the odour source, while the other three jars contained beakers of tap water only and served as controls. The air flow had been visualised and controlled during preliminary tests by means of an air flow indicator (Smokpoint^®^, Wöhler Technik GmbH, Bad Wünnenberg, Germany). Silicone tubing was used for all connections between the different parts of the setup.

The observation zones were delineated on the filter paper at the bottom of the star-shaped arena. Starting from the centre, the four quadrants were divided so that each could be assigned to one of the four arms. Additionally, there was a circular starting zone with a 2 cm diameter positioned exactly in the centre. This circular starting zone was surrounded by a second circle with an 8 cm diameter, which served as the first-choice line for the respective quadrant. In all experiments, quadrant 1 was assigned to the respective odour source, while quadrants 2 (to the left of quadrant 1), 3 (opposite), and 4 (to the right) were assigned to the control glass jars.

At the beginning of an observation of a single male or female, lasting a maximum of 6 min, the weevil was placed in the starting zone through the central hole in the cover plate using a pair of tweezers. The observation time was recorded from the time the silicone stopper with the air suction tube was connected to the cover plate. Entry into an observation zone, i.e., quadrant, was recorded when the weevil had crossed one of the designated boundary lines with its front legs. Weevils that did not leave the starting zone within 3 min were excluded from the experiment. This maximum latency to end the immobility of the beetles after release into the olfactometer had been determined in preliminary tests. The first choice of a quadrant, percentage of entries into each quadrant, and the percentage of time spent by the individuals in each quadrant were recorded using The Observer Video-Pro 5.0 software (Noldus Information Technologies bv, Wageningen, The Netherlands). If a weevil entered one of the connecting tubes leading to the glass jars, the observation was terminated. To avoid positional bias, the olfactometer, along with all the connected jars, was rotated by 90° after every third observation. Each experiment consisted of 12 observations, and each experiment was replicated three times. This means the responses of 3 × 12 females and 3 × 12 males towards the leaf volatiles of each food plant species were recorded. After each experiment, the arena, the glass jars, and the connecting tubing were first cleaned with tap water and then with acetone (≥99.8%) and allowed to dry for at least one hour.

### 2.4. Statistical Analysis

A chi-square goodness-of-fit test (df = 3) was used to compare the weevils’ first choices for each quadrant of the four-arm olfactometer to a theoretical distribution based on equal preferences for each of the quadrants. This test was performed using IBM SPSS Statistics for Windows, Version 28.0. (IBM Corp, Armonk, NY, USA). The null hypothesis of equal time (%) spent in each quadrant and equal frequency of entries (%) into each quadrant was tested using Friedman’s nonparametric two-way ANOVA using R, R version 4.2.3 (R core team, Vienna). *p*-values smaller than 0.05 were regarded significant.

## 3. Results

Significantly more *A. punctiventris* females chose the quadrant, i.e., the odour field with leaf odours of *B. vulgaris* subsp. *vulgaris* (Altissima group) or *B. vulgaris* subsp. *vulgaris* (Cicla group), as their first choice than chose the control quadrants (Table 1). The quadrant with leaf volatiles from *A. hortensis*, *C. album*, or *A. retroflexus* were not the first choice for the females. A significantly higher number of male sugar beet weevils chose the odour field with leaf odours from *B. vulgaris* subsp. *vulgaris* (Cicla group) or *A. hortensis* first compared to the control fields. However, this first choice was not observed with *B. vulgaris* subsp. *vulgaris* (Altissima group), *C. album*, or *A. retroflexus.*

**Figure 1:**
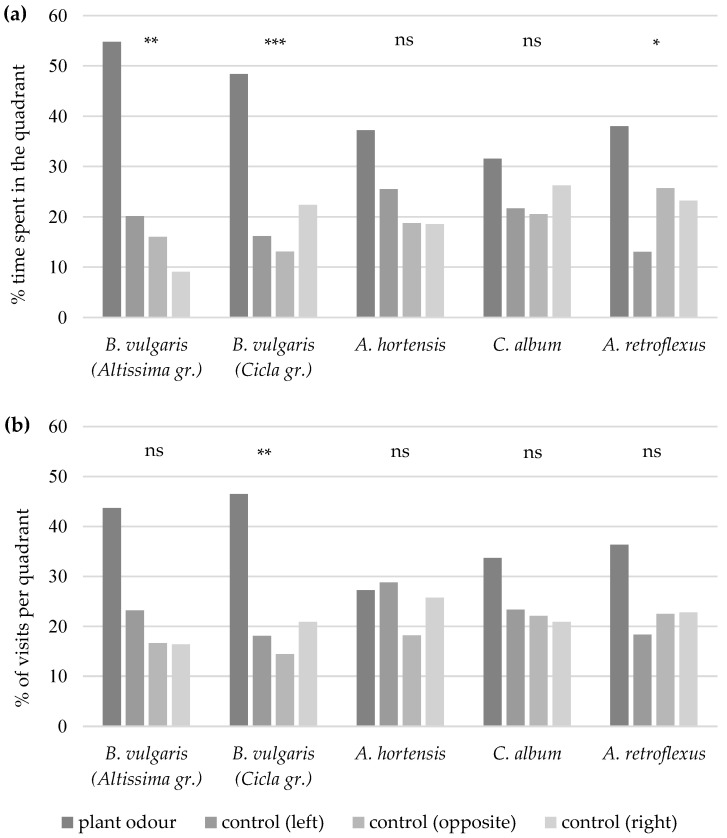
Percentage of time spent (**a**) and percentage of visits (**b**) by *A. punctiventris* females per quadrant with plant odour of *B. vulgaris* subsp. *vulgaris* (Altissima group), *B. vulgaris* subsp. *vulgaris* (Cicla group), *A. hortensis*, *C. album*, or *A. retroflexus* leaves or in the control quadrants (clean air) to the left of, to the right of, or opposite the plant odour quadrant of the 4-arm olfactometer. Asterisks indicate significant differences between quadrants (*p* ≤ 0.05 = *; *p* ≤ 0.01 = **; *p* ≤ 0.001 = ***); non-significant differences (*p* > 0.05) are indicated by ns.

The females of *A. punctiventris* spent significantly more time in the quadrant with the leaf odour of *B. vulgaris* subsp. *vulgaris* (Altissima group) (Friedman χ^2^ = 16.19, df = 3, *p* = 0.001) or *B. vulgaris* subsp. *vulgaris* (Cicla group) (Friedman χ^2^ = 16.78, df = 3, *p* < 0.001) (Figure 1a) and visited this quadrant more frequently than the control quadrants of the four-armed olfactometer (*B. vulgaris* Altissima group: Friedman χ^2^ = 7.19, df = 3, *p* = 0.066; *B. vulgaris* Cicla group: Friedman χ^2^ = 14.65, df = 3, *p* = 0.002) (Figure 1b). Except for *A. retroflexus* (Friedman χ^2^ = 7.88, df = 3, *p* = 0.049), the females did not stay significantly longer in the quadrants with leaf odour from *A. hortensis* (Friedman χ^2^ = 7.18, df = 3, *p* = 0.066) or *C. album* (Friedman χ^2^ = 2.49, df = 3, *p* = 0.477) than in the control quadrants. They also did not enter the odour fields with leaf odours of the latter three plants more frequently than those with clean air as control (*A. retroflexus*: Friedman χ^2^ = 3.45, df = 3, *p* = 0.327; *A. hortensis*: Friedman χ^2^ = 3.73, df = 3, *p* = 0.292; *C. album*: Friedman χ^2^ = 2.57, df = 3, *p* = 0.463).

**Figure 2:**
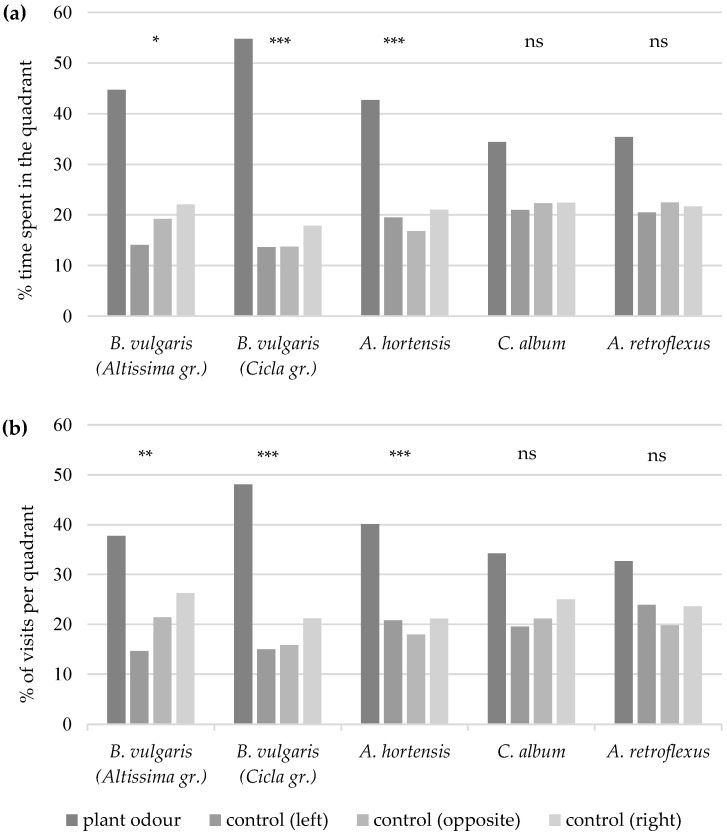
Percentage of time spent (**a**) and percentage of visits (**b**) by *A. punctiventris* males per quadrant with the plant odour of *B. vulgaris* subsp. *vulgaris* (Altissima group), *B. vulgaris* subsp. *vulgaris* (Cicla group), *A. hortensis*, *C. album*, or *A. retroflexus* leaves or in the control quadrants (clean air) to the left of, to the right of, or opposite the plant odour quadrant of the 4-arm olfactometer. Asterisks indicate significant differences between quadrants (*p* ≤ 0.05 = *; *p* ≤ 0.01 = **; *p* ≤ 0.001 = ***); non-significant differences (*p* > 0.05) are indicated by ns.

Male sugar beet weevils spent significantly more time in the quadrant with the leaf odour of *B. vulgaris* subsp. *vulgaris* (Altissima group) (Friedman χ^2^ = 10.39, df = 3, *p* = 0.016), *B. vulgaris* subsp. *vulgaris* (Cicla group) (Friedman χ^2^ = 22.22, df = 3, *p* < 0.001), or *A. hortensis* (Friedman χ^2^ = 11.09, df = 3, *p* < 0.001; Figure 2a) and visited this quadrant significantly more frequent than the control quadrants (*B. vulgaris* Altissima group: Friedman χ^2^ = 12.61, df = 3, *p* = 0.006; *B. vulgaris* Cicla group: Friedman χ^2^ = 26.13, df = 3, *p* < 0.001; *A. hortensis*: Friedman χ^2^ = 19.22, df = 3, *p* < 0.001) (Figure 2). They did not show clear responses to the odours of *C. album* (percentage of time: Friedman χ^2^ = 5.18, df = 3, *p* = 0.159; percentage of visits: Friedman χ^2^ = 4.74, df = 3, *p* = 0.192) or *A. retroflexus* (percentage of time: Friedman χ^2^ = 3.88, df = 3, *p* = 0.275; percentage of visits: Friedman χ^2^ = 5.37, df = 3, *p* = 0.146).

## 4. Discussion

The aim of the present study was to investigate whether *A. punctiventris* uses leaf odours to locate the plants it is feeding on. This required the elaboration of an olfactometer bioassay procedure that takes into account the motionless behaviour of the sugar beet weevil on a plant when threatened by a natural enemy [19]. In our experiments, some weevils showed immobility for about two to three minutes after being placed on the starting zone in the olfactometer with a pair of tweezers, presumably mimicking a predator. We therefore set a time limit of three minutes for the weevils to leave the starting zone to account for this behaviour. Immobility, which is used to reduce the risk of predator detection or tracking, occurs earlier in the sequence of a predatory attack than thanatosis (death feigning) [25]. This behaviour is also exhibited by *A. punctiventris* but lasts only about 15 to 17 s [19]. Nakamuta et al. [26] used a special Y-track olfactometer with a release container for insects showing thanatosis, where the start of the observation period is triggered by the insects themselves climbing up the vertical bar of the Y-track from a release container. However, by using a four-armed olfactometer and setting an appropriate time limit for the weevils in this study, we were able to record three different behavioural parameters. The statistical power in a four-arm olfactometer for a single-quadrant, one-odour test situation is higher than in a Y-track olfactometer [24]. Recording the frequency of visits and time spent by the sugar beet weevils in each quadrant of the olfactometer, combined with their first choice, allowed us to quantify their behaviour and clearly assess their responses to different odours of their food plants.

After leaving their overwintering sites in the soil, sugar beet weevils move randomly on the soil surface for a short time before starting to search for food plants [1,19,20]. The weevils tested in our laboratory experiments were at this stage; after a period of artificial overwintering, they were kept for 2–3 days at a temperature of 20 °C, which was higher than the soil temperature at the overwintering site. Having been collected from winter wheat fields where sugar beets had been grown the previous year, they had no experience of feeding on sugar beets and were hungry when tested in the four-arm olfactometer. Eckstein [1] observed *A. punctiventris* in the field during this period and assumed that it was guided to its food plants by sensory input to its antennae. The odour of fresh green vegetation carried by a moderate wind is a potential source of attraction for the sugar beet weevil, as noted by Auersch [19]. It is not known yet whether and what kind of sensilla for the perception of volatile olfactory stimuli are localised on the antennae of *A. punctiventris*. However, our olfactometer experiments showed, for the first time, that the sugar beet weevils respond to leaf volatiles entrained in moving air. Adult females and males were equally attracted to the leaf odours of young *B. vulgaris* subsp. *vulgaris* plants, i.e., sugar beet and chard, as indicated by the overall picture of the behavioural parameters analysed. Similarly, both sexes of the oligophagous banana weevil (*Cosmopolites sordidus* Germar) responded positively to host plant volatiles, suggesting that the orientation responses observed are probably related to finding food rather than finding oviposition sites [27]. This could also apply to *A. punctiventris*, and the olfactory attraction to *B. vulgaris* subsp. *vulgaris,* the plant with the highest feeding value [5], could be due to the fact that both sexes need to go through a phase of maturation feeding [4,20]. In a leaf consumption comparison of food plants from the family Amaranthaceae, unmated female and male sugar beet weevils were found to consume the largest amounts of leaf mass from sugar beets and chard of all plants tested. In addition, the most and heaviest fourth instar larvae developed on sugar beets [23]. Whether *A. punctiventris* females also prefer *B. vulgaris* to other plants for oviposition remains to be investigated. Such studies should also shed light on the role of leaf odours in the localisation of oviposition sites. When oviposition sites are associated with the host plant, recognising host odours provides an additional evolutionary advantage for the female insect [28]. However, the next step is to determine whether the sugar beet weevil is attracted to specific ratios of compounds commonly found in green leaves. This can be achieved by analysing and testing the volatile components of *B. vulgaris* leaf odours.

Individuals that leave their overwintering sites before sugar beets emerge also feed on wild plants, such as Chenopodium and Atriplex species or *A. retroflexus* [4]. Weevils of both sexes consumed, on average, 30% less leaf mass of *A. hortensis* and, on average, 65% less of *C. album* or *A. retroflexus* than of *B. vulgaris* subsp. *vulgaris.* Larvae can develop on the roots of *A. hortensis* and *C. album*, albeit in smaller numbers than on sugar beets, but not on the roots of *A. retroflexus* [23]. While *A. punctiventris* males, but not females, were attracted to the leaf odour of *A. hortensis* in our olfactometer experiments, no response was observed when the weevils were tested with leaf odours of *C. album* or *A. retroflexus*. This is in spite of the presumed advantage of olfactory recognition of these plants for the females, as there are indications that they could lead to higher egg deposition rates when fed with sugar beets as part of a mixed diet [29].

Auersch [19] describes, without distinguishing between the sexes, how sugar beet weevils, wandering on the ground, touch plants they come across by chance with their antennae and examine them carefully. Antennae are used not only for olfaction but also for taste in many insect taxa, including Coleoptera [30]. The oligophagous Colorado potato beetle (*Leptinotarsa decemlineata* Say) is strongly attracted to the volatiles of potato plants (*Solanum tuberosum* L.) [31,32] but discriminates between preferred and less preferred host plants upon first contact, as expressed by its behaviour of walking, palpating and antennal waving when examining the plants, presumably perceiving sensory input on the sensilla located on the tarsi, mouthparts, and antennae [33]. It is possible that food plant recognition in *A. punctiventris* involves not only olfaction but also contact chemoreception; plants with a feeding value lower than *B. vulgaris* subsp. *vulgaris*, such as *C. album* or *A. retroflexus* [5], might be only accepted upon contact, and acceptance may vary between females and males. In bioassays in petri dishes, Dittmann et al. [23] offered leaves of the latter plants to the weevils, which accepted them as food as they came into contact with them. Again, sensilla on the antennae, mouthparts, and other body parts of this Curculionid species still need to be investigated.

In conclusion, the results of the present study provide the first experimental evidence that adult *A. punctiventris* use leaf odours to locate sugar beet and other food plants. Knowledge on the olfactory responsiveness of this pest provides a basis for the further investigation of leaf volatiles, which could potentially be used to improve monitoring or mass trapping strategies.

## Figures and Tables

**Table 1:** Number of first choices of *A. punctiventris* females or males for the quadrant with plant odour of *B. vulgaris* subsp. *vulgaris* (Altissima group), *B. vulgaris* subsp. *vulgaris* (Cicla group), *A. hortensis*, *C. album*, or *A. retroflexus* leaves or for the control quadrants (clean air) of the 4-arm olfactometer; *n* = 36.

Plant Odour	Number of Females’ First Choices				Number of Males’ First Choices			
	Quadrant with Plant Odour	Control Quadrants 2, 3, 4 (sum) *	*χ* ^2^	*p*	Quadrant with Plant Odour	Control Quadrants 2, 3, 4 (sum) *	*χ* ^2^	*p*
*B. vulgaris* (Altissima gr.)								
	18	6, 5, 7 (18)	12.22	0.007	15	5, 9, 7 (21)	6.22	0.101
*B. vulgaris* (Cicla gr.)								
	17	7, 3, 9 (19)	11.56	0.009	19	6, 5, 6 (17)	14.89	0.002
*A. hortensis*								
	13	6, 7, 10 (23)	3.33	0.343	17	3, 8, 8 (19)	11.33	0.010
*C. album*								
	11	8, 10, 7 (25)	1.11	0.774	15	6, 5, 10 (21)	6.89	0.076
*A. retroflexus*								
	14	5, 7, 10 (22)	5.11	0.164	13	7, 7, 9 (23)	2.67	0.446
							

* clean air.

## Data Availability

The data presented in this study are available on request from the corresponding author.
